# Seroprevalence of *Toxoplasma gondii* infection and variables associated with seropositivity in donkeys in eastern China

**DOI:** 10.1051/parasite/2018066

**Published:** 2018-12-11

**Authors:** Qing-Feng Meng, Dan Li, Gui-Zhe Yao, Yang Zou, Wei Cong, Xiao-Feng Shan

**Affiliations:** 1 Engineering Research Centre of Chinese Ministry of Education for Edible and Medicinal Fungi, Jilin Agricultural University Changchun 130118 PR China; 2 Inspection and Quarantine Technology Center of Jilin Entry-Exit Inspection and Quarantine Bureau Changchun Jilin Province 130062 PR China; 3 College of Animal Science and Technology, Jilin Agricultural University Changchun Jilin Province 130118 PR China; 4 Marine College, Shandong University at Weihai Weihai Shandong Province 264209 PR China

**Keywords:** *Toxoplasma gondii*, donkeys, seroprevalence, eastern China

## Abstract

Donkeys (*Equus asinus*) are widely distributed throughout China; they are used for their meat, as food, and certain donkey-derived items are also important for traditional Chinese medicinal purposes. However, only limited information is available on *Toxoplasma gondii* infection in donkeys in China, especially the eastern region, which is one of the largest production areas. Thus, the present study was conducted to detect specific anti-*T. gondii* antibodies using a commercially available indirect hemagglutination test (IHA) kit and to evaluate the risk factors that are associated with seroprevalence in the Shandong province of eastern China. A total of 213/1278 (17%) donkeys tested from Shandong province were positive for *T. gondii* antibodies. Statistical analysis revealed that gender and feeding habits of the animal are associated with *T. gondii* infection. These results provide information for the prevention and control of toxoplasmosis in donkeys, other animals, and humans in this region and elsewhere.

## Abstract (fr)

Les ânes (*Equus asinus*) sont largement répandus dans toute la Chine ; ils sont utilisés pour leur viande et certains éléments dérivés des ânes sont aussi importants pour la médecine traditionnelle chinoise. Cependant, seules des informations limitées sont disponibles sur l’infection à *Toxoplasma gondii* chez les ânes en Chine, en particulier dans la région orientale, qui est l’une des plus grandes zones de production. Ainsi, la présente étude a été menée pour détecter des anticorps spécifiques anti-*T. gondii* à l’aide d’un kit de test d’hémagglutination indirecte (IHA) commercial et évaluer les facteurs de risque associés à la séroprévalence dans la province du Shandong, dans l’est de la Chine. Un total de 213/1278 (17 %) ânes de la province du Shandong étaient positifs pour les anticorps anti-*T. gondii*. L’analyse statistique a révélé que le sexe et les habitudes alimentaires de l’animal sont associés à l’infection à *T. gondii*. Ces résultats fournissent des informations pour la prévention et le contrôle de la toxoplasmose chez les ânes, les autres animaux et les humains dans cette région et ailleurs.

## Introduction

*Toxoplasma gondii* is a foodborne parasite that causes zoonotic toxoplasmosis and can infect the humans and nearly all the warm-blooded animals [15]. Currently, one third of the global population is estimated to be infected with this parasite [21]. Interestingly, the definitive hosts of *T. gondii* include wild and domestic felids, which can discharge millions of oocysts of the parasites into the environment following primary infection, thereby posing a major public health concern [15]. As a result, toxoplasmosis remains an unsolved public health issue worldwide [8]. The primary routes of *T. gondii* infection in humans are by ingesting raw or undercooked meat from infected animals and consuming food or water contaminated with infected cat feces containing *T. gondii* oocysts [4]. Thus, consumption of meat from *T. gondii*-infected animals (including poultry, pigs, donkeys, sheep, and cattle) plays a major role in the transmission of the parasite [4].

The donkey (*Equus asinus*), a member of the Equine family, has been identified as an intermediate host of *T. gondii* [1–3, 5, 7, 11–13]. According to a previous study, the output of donkey meat is 182,900 tons in China, accounting for about one third of global donkey meat production [20]. In recent years, the demand for donkey meat has risen sharply. A number of studies have been conducted worldwide to detect the prevalence of *T. gondii* infection in donkeys; however, the surveys conducted in China were done several years ago. Although Shandong province is a major donkey breeding area in China, only limited information on *T. gondii* infection in this species is available for this part of eastern China. In recent years, the provincial government has begun to focus on the development of the donkey industry. However, large gaps exist in the prevention and control of parasitic diseases, especially for food-borne zoonotic diseases. Therefore, the present study was conducted to detect the seroprevalence of *T. gondii* infection and to evaluate the variables associated with seropositivity in donkeys in Shandong province. These data would provide valuable information for the prevention and control of toxoplasmosis in donkeys and humans in this region and elsewhere.

## Materials and methods

### Ethics statement

This study was approved by the Animal Ethics Committee of Jilin Agricultural University. Serum samples were collected and handled in accordance with the requirements of the Animal Ethics Procedures and Guidelines of the People’s Republic of China.

### Collection and preparation of serum samples

Serum samples were randomly collected from the jugular vein of 1278 donkeys from various regions in Shandong province (4°23′~38°24′ N, 114°48′~122°42′ E) between July 2015 and December 2016 by local veterinary practitioners. The animals from each farm were selected randomly using a table of random digits. Several large-scale farms (>500 animals) were not included because the owner was not available to obtain permission. Finally, a total of 17 farms were selected, and approximately 30% of donkeys were sampled on each farm. All the sampled animals were clinically healthy. For backyard donkeys, we randomly visited local individual farmers, obtained permission, and collected the blood samples from the animal. Finally, a total of 623 Dezhou donkeys (257 from Heze, 142 from Jining, 224 from Liaocheng), 354 Sanfen donkeys (53 from Heze, 152 from Jining, 149 from Liaocheng), and 301 Wutou donkeys (66 from Heze, 110 from Jining, 125 from Liaocheng) were sampled (Table 1). Donkeys from farms and backyards were commonly used for slaughtering and agriculture, respectively. The serum was obtained by centrifugation of the blood samples at 1000 ×*g* for 5 min and stored at −20 °C until analysis.

**Table 1.** Seroprevalence of *T. gondii* infection in donkeys in Shandong province, eastern China by IHA.

Host	No. of serum samples with IHA titers					No. positive	No. tested	Prevalence (%) (95% CI)
	1:64	1:128	1:256	1:512	1:1024			
Dezhou donkey	41	32	29	9	2	112	623	17.98 (14.96–20.99)
Wutou donkey	16	16	13	3	0	48	354	13.56 (9.99–17.13)
Sanfen donkey	19	14	14	6	0	53	301	17.61 (13.31–21.91)
Total	76	62	56	18	2	214	1278	16.75 (14.70–18.79)

### Serological examination

Antibodies against *T. gondii* were detected using a commercially available indirect hemagglutination test (IHA) kit (Lanzhou Veterinary Research Institute, Chinese Academy of Agricultural Sciences, Lanzhou, Gansu Province, China) (http://lvri.caas.cn/kjpt/zdjczx/index.htm), according to the manufacturer’s recommendations. The method is a national standard (NY/T 573-2002) of China for the detection of animal toxoplasmosis. Briefly, sera were added to 96-well V-bottomed polystyrene plates and diluted two-fold from 1:4 to 1:2048. IHA titers ≥1:64 (manufacturer’s recommendation) were considered positive when forming a layer of agglutinated erythrocytes; sera with dubious results were re-tested. Positive and negative controls, supplied by the Lanzhou Veterinary Research Institute, were included and tested at dilutions identical to those of the serum samples. Consequently, IHA showed 89.8% sensitivity and 96.6% specificity in detecting *Toxoplasma* IgG antibody [18].

### Statistical analysis

The current data were analyzed by the SPSS 18.0 software package (IBM, Armonk, NY, USA). *P*-values <0.05 were considered to reflect a significant difference. Logistic regression was used to analyze the association between *T. gondii* infection and the potential risk factors. The multivariate logistic analysis was performed with the full model including all potential risk factors.

## Results and discussion

In the present study, 214/1278 donkeys (16.75%, 95% confidence intervals (CIs): 14.70–18.79) were seropositive for *T. gondii* (Table 1). The *T. gondii* seroprevalence in Dezhou Donkeys, Wutou Donkeys, and Sanfen Donkeys was 17.98%, 13.56%, and 17.61%, respectively. The highest seroprevalence was found in Liaocheng (17.47%), followed by Jining (16.58%) and Heze (15.96%). The seroprevalence in female and male donkeys was 19.10% and 14.81%, respectively. The seroprevalence among different age groups ranged from 14.01% in the Age > 5 years group to 18.50% in the 1 < Age ≤ 3 years group. Moreover, donkeys bred in the backyard (30.15%) showed a significantly higher seroprevalence than those on the farm (14.27%) (*P* < 0.001) (Table 2). Further analysis using multivariate logistic regression revealed that gender (odds ratio (OR) = 1.350, 95% CI: 1.00–1.82, *P* = 0.049) and feeding habits (OR = 2.572, 95% CI: 1.80–3.67, *P* < 0.001) were risk factors for *T. gondii* seroprevalence.

**Table 2.** Univariate analysis of the variables associated with *T. gondii* seroprevalence in donkeys in Shandong province, eastern China tested by IHA.

Variable	Category	No. tested	Prevalence (%)	OR	95% CI	*P*-value
Breed	Dezhou donkey	623	17.98	Reference		
	Sanfen donkey	354	13.56	0.72	0.50–1.03	0.073
	Wutou donkey	301	17.61	0.98	0.68–1.40	0.891
Age (yr)	Age ≤ 1	274	17.52	Reference		
	1 < Age ≤ 3	400	18.50	1.07	0.72–1.60	0.745
	3 < Age ≤ 5	347	16.14	0.91	0.59–1.38	0.648
	Age > 5	257	14.01	0.77	0.48–1.23	0.268
Region	Jining	404	16.58	Reference		
	Liaocheng	498	17.47	1.07	0.75–1.51	0.725
	Heze	376	15.96	0.96	0.65–1.40	0.813
Gender	Male	702	14.81	Reference		
	Female	576	19.10	1.36	1.01–1.82	0.041
Feeding habits	Farm	1079	14.27	Reference		
	Backyard	199	30.15	2.59	1.83–3.67	<0.001
Total		1278	16.75			

Donkeys are an important animal for traditional Chinese medicinal and comestible purposes and are widely distributed in China [19]. Previous surveys have reported the prevalence of *T. gondii* infection in donkeys in China [14, 17, 20]. However, the present results cannot be compared with those from other studies due to the differences in factors such as the breed of the donkeys, the number of tested animals, age classes, and animal hygiene standards. Furthermore, it is difficult to compare the current and the previous studies due to different serological tests employed.

In the present study, the feeding habits of donkeys were the only variable significantly associated with seroprevalence (*P* < 0.001). Interestingly, the donkeys bred in backyards were more easily infected with *T. gondii* as compared to those bred on farms. During this survey, cats were commonly found in the backyards. Although the infection status of cats was not tested, breeding cats at home might be the primary cause of high seroprevalence in donkeys bred in backyards. Also, other risk factors such as water, food, or pastures contaminated with *T. gondii* oocysts should be considered. Thus, additional studies are essential to substantiate the current findings and identify the optimal method for reducing *Toxoplasma* infection in donkeys.

The seropositivity of *T. gondii* appears to be related to age [6, 16]; however, *T. gondii* seroprevalence in donkeys was not significantly influenced by age. The analysis of age distribution in donkeys demonstrated that most of the backyard donkeys could be categorized in the 1 < Age ≤ 3 years group, which might influence seropositivity. However, further targeted studies are essential to explore the effect of age on *T. gondii* seropositivity with respect to different feeding habits in donkeys. Strikingly, female donkeys showed a significantly higher seroprevalence than male donkeys (*P* = 0.041), and thus, future studies should focus on the impact of *T. gondii* infection in the offspring of donkeys.

In the present study, all farms were positive for the presence of *T. gondii*, rendering it is impossible to evaluate the role of farms as a risk factor. Thus, further studies should be conducted to explore the role of toxoplasmosis in reproductive and economic losses in donkey breeding in these regions.

Several laboratory tests have been used for the detection of *T. gondii* antibodies in donkeys, including the modified agglutination test, ELISA, IHA, and PCR [11–14, 17]. Herein, we selected IHA to detect *T. gondii* antibodies in donkeys because this kit has high sensitivity and specificity and is easily available [18]. Moreover, the IHA kit has been extensively used for detecting specific antibodies to *T. gondii* in horses, pigs, sheep, and other mammals in China for several years [9, 10]. In the present study, we used this kit to detect specific antibodies to *T. gondii* in donkeys. Thus, further studies should be conducted, using different techniques, to confirm the present results.

The current results showed that *T. gondii* infection is common in donkeys in Shandong province, and the parasite is likely to prevail in the tissues of the animals lifelong. Thus, the donkey, at high risk of infection, could act as a transmission route to humans because donkey meat is a standard food in China. Therefore, additional studies are essential to investigate the role of donkey meat in human infections and the pathogenesis of toxoplasmosis in the animal.

## Conclusions

The present survey revealed a 16.75% seroprevalence of *T. gondii* infection in donkeys in Shandong province, eastern China. Feeding habits were identified as the primary risk factor for *T. gondii* infection in donkeys. The present results provide baseline data for designing and evaluating tools for prevention and control, as well as for future studies on *T. gondii* infection in a large population of donkeys in China.

## Competing interests

The authors declare that they have no competing interests.

## References

[1] Alvarado-Esquivel C, Alvarado-Esquivel D, Dubey JP. 2015 Prevalence of *Toxoplasma gondii* antibodies in domestic donkeys (*Equus asinus*) in Durango, Mexico slaughtered for human consumption. BMC Veterinary Research, 11, 6. 25595816 10.1186/s12917-015-0325-9 PMC4301930

[2] Bártová E, Sedlák K, Kobédová K, Budíková M, Joel Atuman Y, Kamani J. 2017 Seroprevalence and risk factors of *Neospora spp*. and *Toxoplasma gondii* infections among horses and donkeys in Nigeria, West Africa. Acta Parasitologica, 62(3), 606–609. 28682771 10.1515/ap-2017-0073

[3] de Oliveira E, de Albuquerque PP, de Souza Neto OL, Faria EB, Júnior JW, Mota RA. 2013 Occurrence of antibodies to *Toxoplasma gondii* in mules and donkeys in the northeast of Brazil. The Journal of Parasitology, 99(2), 343–345. 22924911 10.1645/GE-3210.1

[4] Dubey JP. 2010 Toxoplasmosis of animals and humans, 2nd edn CRC Press: Boca Raton, FL p. 1–313.

[5] Dubey JP, Ness SL, Kwok OC, Choudhary S, Mittel LD, Divers TJ. 2014 Seropositivity of *Toxoplasma gondii* in domestic donkeys (*Equus asinus*) and isolation of *T. gondii* from farm cats. Veterinary Parasitology, 199(1–2), 18–23. 24140163 10.1016/j.vetpar.2013.09.027

[6] Falusi O, French AL, Seaberg EC, Tien PC, Watts DH, Minkoff H, Piessens E, Kovacs A, Anastos K, Cohen MH. 2002 Prevalence and predictors of *Toxoplasma* seropositivity in women with and at risk for human immunodeficiency virus infection. Clinical Infectious Diseases, 35(11), 1414–1417. 12439806 10.1086/344462 PMC3119037

[7] Gennari SM, Esmerini Pde O, Lopes MG, Soares HS, Vitaliano SN, Cabral AD, Pena HF, Horta MC, Cavalcante PH, Fortes KP, Villalobos EM. 2015 Occurrence of antibodies against *Toxoplasma gondii* and its isolation and genotyping in donkeys, mules, and horses in Brazil. Veterinary Parasitology, 209(1–2), 129–132. 25747488 10.1016/j.vetpar.2015.01.023

[8] Innes EA. 2010 A brief history and overview of *Toxoplasma gondii*. Zoonoses and Public Health, 57(1), 1–7. 10.1111/j.1863-2378.2009.01276.x 19744303

[9] Li F, Wang SP, Wang CJ, He SC, Wu X, Liu GH. 2016 Seroprevalence of *Toxoplasma gondii* in goats in Hunan province, China. Parasite, 23, 44. 27762212 10.1051/parasite/2016053 PMC5075832

[10] Luo H, Li K, Zhang H, Gan P, Shahzad M, Wu X, Lan Y, Wang J. 2017 Seroprevalence of *Toxoplasma gondii* infection in zoo and domestic animals in Jiangxi Province, China. Parasite, 24, 7. 28224883 10.1051/parasite/2017007 PMC5327750

[11] Machacova T, Bartova E, Di Loria A, Sedlak K, Mariani U, Fusco G, Fulgione D, Veneziano V, Dubey JP. 2014 Seroprevalence of *Toxoplasma gondii* in donkeys (*Equus asinus*) in Italy. The Journal of Veterinary Medical Science, 76(2), 265–267. 24107428 10.1292/jvms.13-0352 PMC3982808

[12] Mancianti F, Nardoni S, Papini R, Mugnaini L, Martini M, Altomonte I, Salari F, D’Ascenzi C, Dubey JP. 2014 Detection and genotyping of *Toxoplasma gondii* DNA in the blood and milk of naturally infected donkeys (*Equus asinus*). Parasites & Vectors, 7, 165. 24708691 10.1186/1756-3305-7-165 PMC3985582

[13] Martini M, Altomonte I, Mancianti F, Nardoni S, Mugnaini L, Salari F. 2014 A preliminary study on the quality and safety of milk in donkeys positive for *Toxoplasma gondii*. Animal, 8(12), 1996–1998. 25118707 10.1017/S1751731114001980

[14] Miao Q, Wang X, She LN, Fan YT, Yuan FZ, Yang JF, Zhu XQ, Zou FC. 2013 Seroprevalence of *Toxoplasma gondii* in horses and donkeys in Yunnan Province, Southwestern China. Parasites & Vectors, 6, 168. 23742078 10.1186/1756-3305-6-168 PMC3679964

[15] Montoya JG, Liesenfeld O. 2004 Toxoplasmosis. Lancet, 363, 1965–1976. 15194258 10.1016/S0140-6736(04)16412-X

[16] Nowakowska D, Wujcicka W, Sobala W, Spiewak E, Gaj Z, Wilczyński J. 2014 Age-associated prevalence of *Toxoplasma gondii* in 8281 pregnant women in Poland between 2004 and 2012. Epidemiology and Infection, 142(3), 656–661. 23721799 10.1017/S0950268813001179 PMC9151098

[17] Yang N, Mu MY, Yuan GM, Zhang GX, Li HK, He JB. 2013 Seroprevalence of *Toxoplasma gondii* in slaughtered horses and donkeys in Liaoning province, northeastern China. Parasites & Vectors, 6, 140. 23680297 10.1186/1756-3305-6-140 PMC3659062

[18] Yang YX, Chen YK, Wei SJ, Song RH. 2014 Efficiency of three methods for detecting *Toxoplasma* IgG antibody. Chinese Journal of Schistosomiasis Control, 26, 109–110. 24800586

[19] Zhang P, Ma J, Han BY, Liang RQ. 2013 Analysis of the status quo and trend of donkey industry in China. Chinese Abstract Animal Husbandry and Veterinary Medicine, 2013(29), 36–38 (In Chinese).

[20] Zhang XX, Shi W, Zhang NZ, Shi K, Li JM, Xu P, Zhao Q, Du R. 2017 Prevalence and genetic characterization of *Toxoplasma gondii* in donkeys in northeastern China. Infection, Genetics and Evolution, 54, 455–457. 10.1016/j.meegid.2017.08.008 28807857

[21] Zhou P, Chen Z, Li HL, Zheng H, He S, Lin RQ, Zhu XQ. 2011 *Toxoplasma gondii* infection in humans in China. Parasites & Vectors, 4, 165. 21864327 10.1186/1756-3305-4-165 PMC3174123

